# CO_2_ Hydrogenation Catalyzed by Graphene-Based Materials

**DOI:** 10.3390/molecules27113367

**Published:** 2022-05-24

**Authors:** Maria Mihet, Monica Dan, Mihaela D. Lazar

**Affiliations:** National Institute for Research & Development of Isotopic and Molecular Technologies—INCDTIM, 67-103 Donat Street, 400293 Cluj-Napoca, Romania; maria.mihet@itim-cj.ro (M.M.); monica.dan@itim-cj.ro (M.D.)

**Keywords:** CO_2_ methanation, CO_2_ to methanol, CO_2_ to formic acid, CO_2_ Fischer–Tropsch, graphene catalysts, reduced graphene oxide, N-dopped graphenes

## Abstract

In the context of an increased interest in the abatement of CO_2_ emissions generated by industrial activities, CO_2_ hydrogenation processes show an important potential to be used for the production of valuable compounds (methane, methanol, formic acid, light olefins, aromatics, syngas and/or synthetic fuels), with important benefits for the decarbonization of the energy sector. However, in order to increase the efficiency of the CO_2_ hydrogenation processes, the selection of active and selective catalysts is of utmost importance. In this context, the interest in graphene-based materials as catalysts for CO_2_ hydrogenation has significantly increased in the last years. The aim of the present paper is to review and discuss the results published until now on graphene-based materials (graphene oxide, reduced graphene oxide, or N-dopped graphenes) used as metal-free catalysts or as catalytic support for the thermocatalytic hydrogenation of CO_2_. The reactions discussed in this paper are CO_2_ methanation, CO_2_ hydrogenation to methanol, CO_2_ transformation into formic acid, CO_2_ hydrogenation to high hydrocarbons, and syngas production from CO_2_. The discussions will focus on the effect of the support on the catalytic process, the involvement of the graphene-based support in the reaction mechanism, or the explanation of the graphene intervention in the hydrogenation process. Most of the papers emphasized the graphene’s role in dispersing and stabilizing the metal and/or oxide nanoparticles or in preventing the metal oxidation, but further investigations are needed to elucidate the actual role of graphenes and to propose reaction mechanisms.

## 1. Introduction

The increasing concentration of CO_2_ in our planet’s atmosphere due to human activities is viewed as an important factor determining the temperature increase and the subsequent climate changes observed in the last decades. Therefore, tremendous efforts are concentrated to diminish the emissions of CO_2_, and one viable possibility to achieve this goal is to capture and use the CO_2_ produced in the main polluting industry and energy production sectors, instead of releasing it into the atmosphere. The possibility of transforming the captured CO_2_ into useful and valuable products would only encourage its capture. Potential routes for CO_2_ valorization are its transformation into hydrogenated organic compounds: methane, methanol, formic acid, synthetic fuels, etc. The main problems with the actual possibility to transform CO_2_ in such compounds are the availability of green low-cost hydrogen and the low efficiency of the hydrogenation processes. These drawbacks are reflected in high production costs, and consequently in the economic inefficiency. Hydrogen supply can be achieved using the electricity generated from renewable sources (wind, solar, wave) at the peak production and low consumption, ensuring thus the valorization of temporary excess in power generation (power-to-gas concept). In this way, the short-term and seasonal variations in renewable power generation are equilibrated increasing the process efficiency. The goal of low-cost green hydrogen production is thus achievable by using lower-cost renewable energy. The low efficiency of CO_2_ hydrogenation can be addressed by using new catalysts and by technological development.

The availability of active and selective catalysts for CO_2_ hydrogenation brings an important contribution to increasing the process efficiency, therefore this research area was very active in the last 10–15 years. The high stability of the CO_2_ molecule is the most important challenge in any of the CO_2_ hydrogenation processes, which may be overcome only by the use of an active catalyst. Therefore, a catalyst that can adsorb and activate both CO_2_ and H_2_ molecules is highly desirable to favor their chemical transformation into hydrogenated products. The selection of the catalysts for CO_2_ hydrogenation depends on the desired process: the catalyst composition alongside the reaction conditions determines the selectivity of the process for one or the other hydrogenated products. The CO_2_ hydrogenation reactions that are reviewed in this paper are as follows:

CO_2_ + 4H_2_ → CH_4_ + 2H_2_O        methanation, (1)

CO_2_ + 3H_2_ → CH_3_OH + H_2_O         methanol synthesis, (2)

CO_2_ + H_2_ → HCOOH         formic acid synthesis, (3)

CO_2_ + H_2_ → CO + H_2_O         reverse water gas-shift, (4)

CO_2_ + nH_2_ → hydrocarbons CO_2_         Fischer–Tropsch. (5)

Catalysts used for CO_2_ methanation are mainly oxide-supported ones with known reforming properties [1], while for CO_2_ transformation into methanol, oxide-supported silver or copper-zinc catalysts are used, similar to the ones used in methanol synthesis from syngas [2,3]. The direct transformation of CO_2_ into higher hydrocarbons (olefins, aromatics, synthetic fuels) is a relatively new direction, catalysts investigated so far being either based on zeolites [4], or iron/oxides and iron/carbon [5]. CO_2_ hydrogenation to formic acid (FA) using heterogeneous catalysts is also a relatively new direction, with MOF-supported catalysts proving to be active for this process [6].

**Figure 1:**
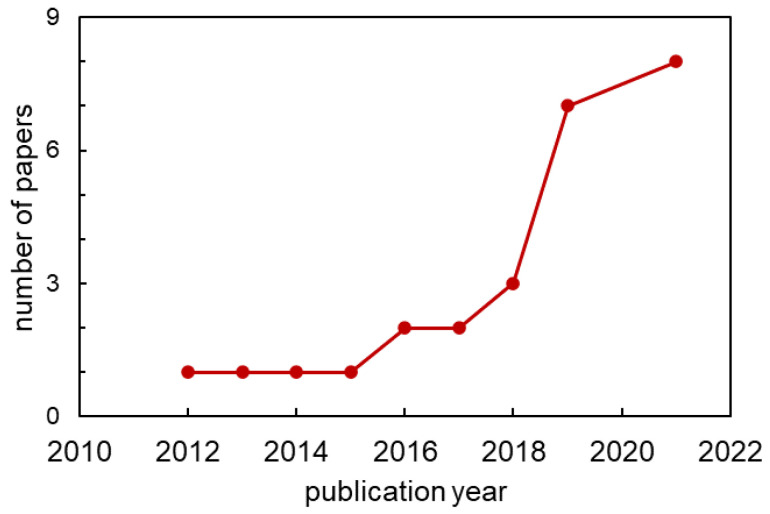
The evolution of the number of published papers reporting studies on CO_2_ hydrogenation catalyzed by graphene-based materials.

The interest in few layers graphene-based materials as catalysts for CO_2_ hydrogenation has significantly increased in the last years (Figure 1) and a dedicated review paper has not yet been published. By the general term of graphene catalysts, we will refer in this paper to few layers graphene oxide (GO), few-layers reduced graphene oxide (rGO), or N-dopped reduced graphene oxide (N-rGO), used either as a catalyst support or as a metal-free catalyst. GO contains up to 10 carbon layers and a large number of oxygenated functional groups on its surface and can be obtained by chemical or electrochemical methods [7]. By chemical or thermal reduction, GO is transformed into rGO, which presents a more ordered graphitic carbon structure but still possesses a limited number of oxygenated functional groups and defects in the carbon layers. GO can be used as a catalyst only at low temperatures, below the thermal reduction that occurs at about 250 °C. Although graphene catalysts were less studied for CO_2_ hydrogenation as compared to the oxide or zeolite-supported ones (mentioned above), the results reported up to now are very interesting and promising for future investigations. In most cases, the catalytic performances are superior to the ones reported for oxide-supported catalysts, either in terms of activity, or, more often, in terms of selectivity and catalyst stability. The advantages of graphene catalysts for CO_2_ hydrogenation processes derive, on one hand, from their carbon composition, and, on the other hand, from the well-known original properties of these 2D carbon materials (high chemical and thermal stability, high thermal conductivity, accessible doping with nitrogen to induce basic sites for CO_2_ activation, and the presence of moderate concentration of defects, which help the dispersion and stabilization of metal or oxide nanoparticles). It is also worth mentioning the possibility to prepare rGO at a relatively low cost and at a large scale through the graphite – graphite oxide – graphene oxide – reduced graphene oxide route. Very interesting catalytic properties were reported for graphene supports with 3D structure, also known as graphene aerogels or porous graphenes. These materials combine the advantages of 2D graphene structure of the walls with the micro, mesoporosity, and high surface area induced by the 3D structure [8].

This review paper will present the catalyst formulations, as well as their performance in the above-mentioned hydrogenation reactions, with emphasis on: (i) the preparation method where this information is significant (i.e., multicomponent catalysts used for methanol synthesis); (ii) the comparison with other published results, if this information is available in the reviewed papers; and (iii) the involvement of the graphene-based support in the reaction mechanism, or the explanation of the role of graphene in the hydrogenation process, in the particular cases when these aspects were studied.

## 2. CO_2_ Hydrogenation to Methane

CO_2_ hydrogenation to methane, also known as CO_2_ methanation, is one of the most studied reactions in the last decade and is very close to up-scaling and technologization for its use on a large scale. CO_2_ methanation is part of the power-to-methane concept [9,10], and the resulting synthetic methane is seen as a promising way for green hydrogen storage and utilization. The advantage would be the use of the existing infrastructure for natural gas transportation and utilization, while the major disadvantage is determined by the efficiency issues raised by the production of synthetic methane. Alongside the technology developments, the existence of highly active and selective catalysts contributes to overcoming these issues. The vast majority of catalysts studied for the methanation of CO_2_ are of metal/oxide type and are regularly revised. The aim of this section is to present the results published up to now on the use of graphene-based catalysts, with emphasis on the role of the support in the catalytic process.

The effect of rGO support and CeO_2_ promotion on Ni catalysts for CO_2_ methanation was studied by comparing the results obtained on Ni/CeO_2_-rGO with the ones obtained on a catalyst bearing the same active metal and promoter, but a different carbon support (i.e., activated carbon, Ni/CeO_2_-AC), as well as with the ones obtained on a classic Ni/CeO_2_-Al_2_O_3_ catalyst [11]. All catalysts were similarly prepared by impregnation/co-impregnation of rGO obtained by hydrazine reduction of GO, and were tested under the same conditions (Table 1). The best results in terms of both CO_2_ conversion and CH_4_ yield were obtained for Ni/CeO_2_-rGO (CO_2_ conversion of 84.5%, compared to 78% for Ni/CeO_2_-Al_2_O_3_, and 59% for Ni/CeO_2_-AC, at 350 °C). In order to discriminate between the support and the promoter influence, catalysts without CeO_2_ were also prepared and tested. For these catalysts, CO_2_ conversion at 350 °C decreased in the series: Ni/rGO(79%) > Ni/Al_2_O_3_(65%) > Ni/AC(38%). It is important to notice that the unpromoted Ni/rGO catalyst also behaved very well compared to the other catalysts considered in this study, its performance being similar to that of Ni/CeO_2_-Al_2_O_3_, while superior to the others. The influence of the graphene support was explained by the authors, considering two aspects: the size and shape of the support surface, and the presence of surface oxygenated functional groups. Catalysts deposited on rGO have a higher surface area than Ni/Al_2_O_3_, but much lower than those deposited on AC (321 m^2^/g compared to 117 m^2^/ and 633 m^2^/g, respectively). Opposite to alumina, both rGO and AC have surface oxygenated functional groups that act as nucleation and anchor centers for the metal nanoparticles, contributing to the dispersion and stability of Ni on the surface. The authors concluded that the enhanced performance of rGO support compared to alumina is due to its higher surface area, and the presence of surface oxygenated groups. In spite of AC’s higher surface area, the enhanced catalytic properties of rGO support compared to AC were attributed to the lower NiNPs size, and, more importantly, to their emplacement on the large, curled graphene sheets. This provides better access of the reagents to the catalytically active sites compared to Ni/AC, in which the larger NiNPs are most likely blocked inside the pores.

Ridzuan et al. prepared Ni/rGO catalysts with three metal loadings (10, 15, and 20 wt.%) by impregnation of commercial rGO and tested these catalysts for CO_2_ methanation [12]. According to the provided characterization results, the employed commercial rGO has approximately 36 carbon layers, which places this material outside the range of few layers graphene (FLG, commonly accepted to have up to 10 graphitic layers), and more to low-layered graphite. The reaction was carried out in liquid phase (dodecane) in a slurry reactor at 10 bar, which is an unusual set-up for CO_2_ methanation. The catalytic activity results showed that the best catalyst in terms of methane productivity was Ni(15%)/rGO, but the best TOF was obtained for Ni(10%)/rGO. By comparing these results with other data from the literature, the authors concluded that Ni(15%)/rGO is a promising catalyst that shows decent CO_2_ conversion values, and very high methane selectivity at lower temperatures compared to oxide-supported Ni catalysts.

Ni impregnated on graphene supports with 3D structure were also prepared and investigated for the methanation of CO_2_ [13,14]. FLG connected by carbon nanofibers (CNF) gives rise to a mesoporous 3D carbon structure with very good properties to host and stabilize the NiNPs [13]. Several parameters, including CNF synthesis conditions (which lead to CNF with varying diameters), as well as different Ni loadings, were tested. The material with the best structural and catalytic performances was the one with a 10 wt.% Ni loading, and the CNF synthesized by chemical vapor deposition (CVD) at 800 °C. The catalysts were tested in two heating arrangements: inductive heating (which heats the magnetically active catalysts), and conventional electric heating. It was proved that inductive heating presents some major advantages for the samples with lower Ni loading (10 wt.%). Higher Ni content generates larger NiNPs, which cause inhomogeneous heating distribution. Inductive heating combined with FLG’s good thermal conductivity provides both (i) lower temperatures for the reaction to take off; and (ii) uniform heating of the catalyst bed, avoiding thus the formation of local heat gradients. This heating mode can be an important advantage at a large scale, where, in the classic heating mode, a large part of the energy is lost to heat the reaction unit alongside the catalyst bed. The selected catalyst leads to high CO_2_ conversion and almost total selectivity for CH_4_ at low temperature (360 °C), also being stable for 100 h time on stream (TOS). The positive role of the graphene support was explained by its: (i) 3D structure with CNF of 17–23 nm diameter, which provides high surface area and a high number of edge sites for anchoring and stabilization of small NiNPs; and (ii) the high thermal conductivity of FLG, which is essential for inductive heating.

Ni deposited on graphene aerogel (Ni/GA) was prepared by heating an aqueous mixture of GO, nickel nitrate, and ammonia in an autoclave at 180 °C [14]. After the reduction in H_2_, the resulting catalyst has a 3D structure and evenly dispersed NiNPs of about 8–12 nm. The tests performed in the methanation of CO_2_ revealed good catalytic activity results, in the same range as for the Ni/CNF-FLG catalyst, and better than the Ni/rGO catalysts with 2D structure (Table 1). In situ DRIFT measurements indicate the formation of carbonate and formate intermediates, suggesting the formate pathway of the reaction mechanism.

A metal-free catalyst for CO_2_ methanation was obtained by impregnation of N-dopped graphene quantum dots (NGQDs) on Al_2_O_3_ [15]. Although the NGQDs have different properties than 2D graphene flakes, it appeared interesting to mention this work here due to the study of the N dopant influence on the hydrogenation of CO_2_. The conclusions can also be extended to N dopped graphene catalysts. GQDs/Al_2_O_3_ (graphene quantum dots with no N content), with very similar structural properties in terms of thickness, size, and crystalline structure was prepared and tested in identical conditions (Table 1) as NGQDs/Al_2_O_3_ and showed no catalytic activity for CO_2_ methanation at any tested temperature. The small amount of converted CO_2_ (2%) at 400 °C gave rise to only CO, proving that no hydrogenation of CO_2_ took place on this material. For NGQDs/Al_2_O_3_ instead, conversion of CO_2_ starts at 180 °C and reaches 65% at 400 °C. The reported CH_4_ selectivity was about 55%, the rest of the reaction products being CO. As explained by earlier reports, the presence of N atoms in carbon materials induces point defects that can delocalize the π bonds of graphenes, creating Lewis basic sites [16,17], which would enhance the adsorption of the acidic CO_2_ molecule. This improvement was also observed for NGQDs/Al_2_O_3_, which explained its catalytic activity for CO_2_ methanation, in contrast to GQDs/Al_2_O_3_. Using N-dopped graphenes with N atoms situated in different positions in the graphene structure, the authors demonstrated that not only the presence but also the position of N dopant significantly influences the activity. The pyridinic N situated at the edge of the graphenes was identified as the catalytic active site for CO_2_ methanation. In the case of NGQDs/Al_2_O_3_, temperature-programmed desorption of CO_2_ (CO_2_-TPD) and IR studies demonstrated that the Lewis basic sites created by pyridinic N not only adsorb but also activate CO_2_ to form COOH^*^, which is the same intermediate as the one proposed on the Ni surface.

## 3. CO_2_ Hydrogenation to Methanol

Methanol is one of the most used chemicals in the modern economy, having important applications in the chemical industry, fine synthesis, and biodiesel production. In the last years, it has also been regarded as a chemical with high potential in the energy sector, either in direct methanol fuel cells [18] or as transportation fuel [19]. Methanol is currently obtained from syngas using commercial alumina-supported Cu-ZnO catalysts. Its direct synthesis from CO_2_ is a very appealing perspective, and great research efforts have been devoted lately in this direction by focusing on either the catalysts [20] or the catalytic processes [21]. Using oxide-supported catalysts, which usually contain a large variety of promoters besides Cu and ZnO, CO_2_ conversions in the range of 5–20%, and CH_3_OH selectivity up to 80% were reported [22]. The advantages of carbon as catalytic support for direct CO_2_ hydrogenation to methanol were recently presented in a review paper [23]. Several forms of carbon, both amorphous (activated carbon) and crystalline (carbon nanotubes, carbon nanofibers, and few layers graphene) were reviewed and discussed. In the present paper, we will only mention in Table 2 the results involving the graphene-supported catalysts already discussed in [23], and will dedicate this section to the new results, with special attention to the discussions regarding the role of graphene in the reaction mechanism.

All studies reviewed here on the hydrogenation of CO_2_ to methanol using graphene-based catalysts reported catalysts composed of Cu-ZnO as the active phase and various graphene materials as supports. Some of them contain FLG or rGO as support [24,25], others N-dopped graphene [26,27,28,29], and others were performed on oxide-graphene composite materials (the oxide being Al_2_O_3_ [28,30], ZrO_2_ [31], ZrO_2_-Al_2_O_3_ [32]). One recent study presents the results obtained using only Cu-Ni bimetallic active phase, with no ZnO, deposited on graphene derivatives (rGO and N-rGO) [33]. Considering that all of the above-mentioned catalysts contain multiple components, the preparation methods were very diverse (Table 2), each one aiming to obtain materials with the optimum interaction between CuO, ZnO, the third oxide, and graphene. The first study concerning CO_2_ hydrogenation to methanol catalyzed by graphene-containing materials was published in 2014 and used CuO-ZnO-Al_2_O_3_-rGO [30]. The catalyst was prepared by mechanically mixing rGO and CuO-ZnO-Al_2_O_3_ mixed oxides (prepared by coprecipitation). The intimate connection between the two components was achieved by ball-milling of the solid mixture. CuO-ZnO-ZrO_2_-Al_2_O_3_-rGO was also prepared by mixing GO with CuO-ZnO-ZrO_2_-Al_2_O_3_ mixed oxides, but in liquid phase (N-methyl-pyrrolidone) [32]. The simultaneous reduction of CuO and GO with ascorbic acid was then achieved.

The co-precipitation of mixed oxides in the presence of graphenes, followed by calcination, was used to prepare CuO-ZnO-Al_2_O_3_-N-rGO [28] and CuO-ZnO-ZrO_2_-rGO [31]. In the first case, the coprecipitation of oxides precursors was made in the presence of N-rGO. In the last case, the simultaneous thermal reduction of oxides and GO was then employed after coprecipitation.

**Figure 2:**
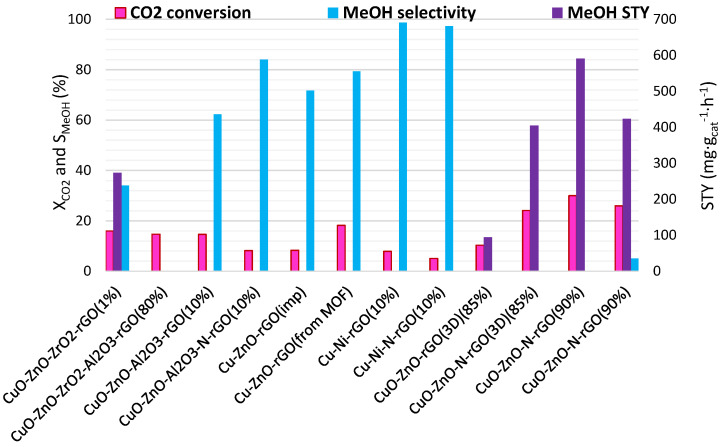
The results of CO_2_ hydrogenation to methanol catalyzed by graphene derivative supported catalysts (data from references [24,25,26,27,28,29,30,31,32,33]).

Impregnation of graphene derivatives (GO, rGO or N-rGO) with Cu(NO_3_)_2_ and Zn(NO_3_)_2_, followed by calcination in air was used to prepare CuO-ZnO-rGO(3D) [24], and CuO-ZnO-N-rGO with bidimensional (2D) [26,27], or three-dimensional (3D) [29] structure. The same impregnation method was used to prepare Cu-ZnO-rGO, but in this case, in order to obtain the copper reduction in the presence of graphene, the calcination was performed in nitrogen at 800 °C [25]. By impregnation of rGO and N-rGO with Cu(NO_3_)_2_ and Ni(NO_3_)_2_, followed by the reduction in (H_2_ + N_2_) (5%H_2_), at 300 °C, a bimetallic Cu-Ni catalyst, with no ZnO, was prepared [33]. The reduction temperature is too low to reduce Ni, or even to fully reduce Cu, therefore the resulting catalyst is a mixture of metallic and oxidic Cu and Ni deposited on the reduced graphene oxide support. For this unusual catalyst, CO_2_ conversion at medium temperatures (225 °C) and high pressures (40 bar) was low, but methanol selectivity is among the highest reported up to now (Figure 2).

A very recent paper reports an interesting method to obtain highly dispersed Cu on graphene [25]. The method denominated by the authors as TGI, “template genetic inheritance”, consists of the synthesis of the Cu containing metal-organic framework (MOF) named HKUST-1 on the graphene surface, followed by its thermal decomposition to gradually generate CuO, and then Cu. Zn(NO_3_)_2_ was also added in the preparation procedure in order to obtain ZnO, the final catalyst being Cu-ZnO-rGO. The role of graphene was to provide a large surface area for the dispersion of HKUST-1, but also to act as the carbon source for the in-situ CO reduction of CuO formed after MOF decomposition. A reference catalyst with similar composition, obtained by impregnation, and thermally treated in identical conditions, was also prepared.

Figure 2 presents the best CO_2_ conversion and methanol selectivity values reported for the graphene-supported catalysts. Due to important differences in the composition of the catalysts, or material preparation and testing conditions, a direct comparison of these results cannot be made, but the information in Figure 2, corroborated with Table 2, offers a general image of the activity of graphene supported catalysts. The first observation is that some of these materials show improved CO_2_ conversion, and especially significantly better selectivity for methanol formation than the classic oxide supported catalysts usually employed for CO_2_ hydrogenation (for which conversions of 5–20% and CH_3_OH selectivities of up to 80% were reported [22]). The second observation is that nitrogen-doped graphene supports provide better catalytic results in the hydrogenation of CO_2_ to methanol than the corresponding undoped carbon materials. The explanation for this effect is that, besides the enhancement of CO_2_ adsorption and H_2_ dissociation (similar to other CO_2_ hydrogenation processes discussed in the previous section), the N species (especially in the form of pyridinic-N) also promote the reduction of CuO [29]. In addition, pyridinic-N can attract hydrogen donor molecules with a positive effect in the reaction path of CO_2_ conversion to methanol [26]. The third observation is that CO_2_ conversion and CH_3_OH selectivity values obtained on the Cu-ZnO-rGO prepared from MOF were significantly better as compared to the one obtained by impregnation (Figure 2). The authors attributed these results to the synergistic effect of graphene and HKUST-1, which improved the: (i) surface area, (ii) Cu reducibility; (iii) adsorption capacity of H_2_ and CO_2_, but no quantitative measurements of these parameters were provided in the paper to sustain these affirmations.

Regarding the involvement of the graphene support in the mechanism of CO_2_ hydrogenation to CH_3_OH, two papers discussed this aspect and concluded that:

1. N-rGO in CuO-ZnO-Al_2_O_3_-N-rGO catalysts plays both the support and promoter role [28]. As support it contributes to the dispersion of Cu, while as the promoter, graphene forms a bridge between the oxide and Cu, thus enhancing the transfer of activated species to meet each other. Its presence improves the spillover of H^*^ from the Cu surface to the Cu-ZnO interface, where the activation of CO_2_ takes place. The basic sites of N-rGO improve both CO_2_ adsorption and its transfer to the Cu-ZnO interface.
2. In the case of CuO-ZnO-ZrO_2_-rGO a similar dual-site mechanism is proposed, in which rGO acts as a bridge between the Cu surface and the surfaces of the oxide (ZnO and ZrO_2_), which are not directly connected to the metal [31]. This is possible by the promotion of hydrogen spillover from Cu to meet not only the activated CO_2_ species (mostly formate) situated at the Cu-oxide interface but also the ones adsorbed on the isolated oxide nanoparticles. In this way, more catalytic active sites are effectively used in the hydrogenation process compared to the rGO free catalyst in which these isolated sites for CO_2_ adsorption cannot meet the activated hydrogen, and consequently are not possible to be involved in the reaction.

## 4. CO_2_ Hydrogenation to Formic Acid (FA)

**Scheme 1:**
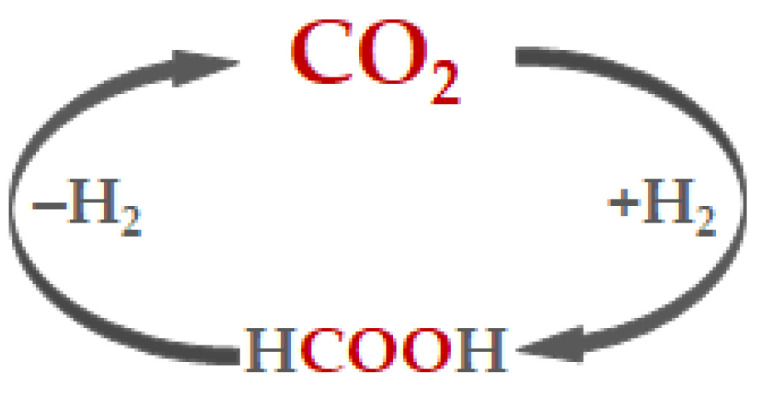
Formic acid as a liquid carrier for hydrogen storage.

The CO_2_ transformation into FA was developed in the last years as a promising alternative in the quest for new possibilities to store hydrogen in liquid carriers. The concept is based on the cycle of FA formation from CO_2_ and H_2_ (on-site, at the hydrogen production facility), followed by FA decomposition in CO_2_ and H_2_ (also named dehydrogenation), when and where hydrogen is needed (Scheme 1).

In this way, CO_2_ is captured and recycled, while the advantage of such a storage alternative is the much simpler and safer manipulation (storage, transportation, etc.) of a stable, biodegradable, and low toxic liquid (FA) than of gaseous hydrogen. The disadvantage comes with the energy consumption associated with the formation and dehydrogenation of FA. Therefore, the main research focus is to improve the efficiency of both catalytic processes—FA formation and FA dehydrogenation—in order to increase the final hydrogen recovery with the minimum possible costs.

The formation of pure FA from CO_2_ faces some important challenges, its thermodynamics being unfavorable even at high temperatures and pressures [34]. In order to improve the conversion of CO_2_ to FA, the reaction can be performed in a basic environment when the actual transformation is from HCO_3_^–^ to HCOO^–^. In this case, the reaction product is a salt, so an additional step is required for the recovery of FA. No matter the use of a basic environment or not for the synthesis of FA from CO_2_, the presence of highly efficient catalysts is needed in order to obtain high conversions under mild conditions. CO_2_ hydrogenation to FA, in the absence of bases and using a heterogeneous catalyst, presents a series of important advantages such as the direct formation of FA, avoiding thus the additional transformation steps, the simple separation of the catalyst after reaction, and the use of non-toxic reagents.

There are only a few published papers on graphene-supported catalysts for CO_2_ hydrogenation to FA, but the reported results are very promising. An interesting carbon nanotube–graphene composite material (CNT-rGO) was used as support for Pd, Ni, and PdNi alloy nanoparticles, giving a very efficient catalyst for pure FA preparation [35]. The CNTs and GO in equimolar combination were mixed and ultrasonicated to give rise to a mesoporous material in which the nanotubes and graphene act as a spacer for each other, preventing thus the staking and bundling processes that naturally occur and usually lead to important loss of surface area. The active metals were deposited by impregnation, followed by simultaneous reduction of graphene oxide and metal ions by hydrazine hydrate at 90 °C. The results obtained in CO_2_ hydrogenation to FA are very good, being comparable in terms of turnover number (TON) and turnover frequency (TOF) to the ones obtained in homogeneous catalysis. The best FA yield was obtained at 40 °C and 40 bar. The proposed reaction mechanism emphasizes the role of the Pd_3_Ni_5_ bimetallic system, in which the electron transfer occurs from Ni to Pd, creating electron-deficient and electron-rich states. H_2_ is dissociatively adsorbed on Pd, while CO_2_ is adsorbed on two adjacent Ni sites through both O atoms. Activated H atoms gradually migrate and bond, first to C atoms, and then to one of the O atoms bonded to Pd, forming HCOOH. In this mechanism, the role of the graphene support was assumed to be only to expose the metallic active sites on the surface, thus facilitating their interaction with the reagents.

PdAu bimetallic catalyst deposited on rGO functionalized with phenylenediamine (Pd_x_Au_(1-x)_/PDA-rGO) was used for CO_2_ hydrogenation to FA in basic conditions [36]. Practically, in this case, the reaction was carried out by starting from an aqueous solution of KHCO_3_ in the H_2_ atmosphere in an autoclave, the reaction product being HCOOK. The Pd_0.5_Au_0.5_/PDA-rGO catalyst sample was used to optimize several reaction parameters such as temperature, pressure, reaction time, catalysts amount, and bicarbonate solution concentration. The results indicate the very good activity of this catalyst, especially at low temperatures (close to room temperature), which is an essential characteristic required for the practical applications for hydrogen storage. The potassium formate (PF) formation yield, defined in the above-mentioned paper as KHCO_3_ conversion, is strongly dependent on temperature only in the first hours of reaction. After 2 h of reaction, the PF yield is 55% at 30 °C and almost 90% at 80 °C. After 8 h reaction time, the PF yield at 30 °C reaches 85%, while the yield at 80 °C remained unchanged. The KHCO_3_ conversion is slightly improved by raising the pressure from 1 MPa to 3 MPa and remained unchanged above this pressure. The recyclability of this catalyst is poor due to the instability of PDA on the graphene surface at high H_2_ pressure. For comparative reasons, Pd_0.49_Au_0.51_/rGO catalyst was prepared and tested under similar conditions. The lower PF yield obtained using this catalyst compared to the amine-functionalized one (85% compared to 94% after 16 h at 50 °C and 5 mPa) was explained by the larger dimensions of the bimetallic PdAu NPs (Table 3), but the surface basicity induced by the amine function cannot be excluded. Three times recyclability of this catalyst is very good, with an almost unchanged yield being observed. This emphasizes the important role of the amine function in the catalytic process, but a mechanism was not proposed in this paper. In addition, the involvement of graphene support in the catalytic reaction was not discussed.

Many more papers presenting the results of DFT studies of the hydrogenation of CO_2_ to FA on graphene-supported catalysts were published in the last years compared to papers containing experimental results. The studied catalytic systems were Cu single atom on Gr [37], Ti dopped Gr [38], Cu and Ru single atoms on Gr [39], Co single atom on N-dopped Gr [40], Pt cluster on Gr [41], Cu, Ru, and Pd single atoms on Gr [42]. The theoretical approach is used to identify the most probable elementary steps of the mechanism, to understand the structure of the transition states along the catalytic path, but also to identify the possible role of the graphene support. These papers emphasize thus the involvement of graphene in the catalytic process, more than the experimental studies. It was demonstrated that various defective sites on the graphene surface play important roles: (i) to immobilize the metal atoms on the surface in order to obtain high (or even atomical) dispersion, (ii) to coordinate and/or split H_2_ and CO_2_ molecules; and (iii) to host the transition states. Different reaction mechanisms dependent on the catalysts’ composition were proposed, from which two examples will be presented further. The first example refers to a Cu single atom–Gr catalyst, for which the following mechanism is proposed [37]: (i) heterolytic splitting of H_2_ into hydride coordinated by the Cu atom and proton coordinated to a defective C of graphene created near the Cu atom, (ii) the insertion of CO_2_ into the Cu-H species forming HCOO-Cu/H-Gr intermediate, (iii) the dissociation of a second hydrogen molecule on HCOO-Cu species, followed by the formation of FA rather than the direct protonation of HCOO-Cu with the hydrogen originating from the H-C^*^ (the hydrogenated site of graphene). This mechanism was also proposed by another study [39] and was explained by the strong bond of hydrogen to the defective graphene. In the case of a Ru single atom–Gr catalyst instead, the transitional nature of this metal with partially filled *d*-states induces a much stronger interaction of the metal with the adsorbed hydrogen, changing the reaction mechanism as compared to Cu [39]. In this case, H_2_ is adsorbed and dissociated into atoms on the Ru atom. No active site is created on the nearby C atom to bond hydrogen. The first hydrogen from the Ru atom reacts with CO_2_ forming HCOO intermediate state, which further reacts with the second hydrogen atom and desorbs as FA. It is notable that the different nature of the metal atom induces a different involvement of the graphene support in the catalytic process through a different H_2_ adsorption and activation path.

## 5. Direct CO_2_ Transformation into High Hydrocarbons

The transformation of synthesis gas (CO + H_2_) into hydrocarbons with a high carbon chain is a well-established industrial process known for almost 100 years. Practically, it is a collection of reactions, the best known being the Fischer–Tropsch process (F–T), which produces a variety of high hydrocarbons (mostly alkanes and alkenes), or oxygenated hydrocarbons. The prospect of using CO_2_ alongside, or even instead of CO in this process is a promising possibility of CO_2_ utilization to generate more valuable and more needed chemicals than CH_4_ and CH_3_OH. The process is not simple, and an earlier study demonstrated that, under actual F–T conditions using Co and Fe based catalysts, the reaction products are very different due to increased reaction selectivity for light saturated hydrocarbons instead of higher hydrocarbons [43]. Under these circumstances, the development of active and selective catalysts is of utmost importance, and the advantages of a graphene support were studied in the case of Co/rGO with 3D structure for F–T [44], FeK/rGO with 3D structure for light olefin production [45], and FeK/rGO(3D)-zeolite for aromatics production [46]. In the case of Co/rGO(3D), the aim of the preparation method was to obtain catalysts with three-dimensional porous graphene as support and hexagonal metallic Co as the active phase. For this, cobalt acetylacetonate (Co(acac)_2_) was mixed with GO to take advantage of the metal cation tendency to bond with the oxygenated functional groups of GO, and disperse on its surface. DMF was used both as a solvent and reducing agent at high temperatures under solvothermal conditions. The material with desired 3D reticular structure and hexagonal phase of the metal was obtained after solvothermal reduction at 220 °C for 12 h. Under these conditions, no unreduced GO was detected, and the wire-like Co nanocrystals cover both sides of the rGO layer. For the sample prepared at 180 °C, cubic Co and some GO are present, while for the sample prepared at 200 °C a mixture of both Co phases was detected. The other properties of the catalysts, such as CoNPs size (around 20 nm), and surface area (S_t_ around 130 m^2^/g) were very similar for all samples. CO_2_ conversion to a mixture of C_1_-C_5_ paraffins and olefins is significantly better for the sample prepared at 220 °C, compared to the other two samples (12% compared to ~5%, and ~2% at 300 °C, respectively). These results were also compared with other previously published data, using Co deposited on oxidic supports tested in similar conditions. The CO_2_ conversion obtained using Co/rGO(3D)(220 °C) is significantly better than any of these. The authors attribute this improved performance to the presence of hexagonal-phase Co crystals with high-energy facets and defects, which are “assembled by the thin rGO layers on a more appropriate scale” [44]. The graphene support is also involved in the explanation of the good stability of this catalyst by providing reducing surroundings for CoNPs, preventing thus metal oxidation by the O atoms dissociated from CO_2_. As no comparison with similar hexagonal-phase Co crystals deposited on other carbon supports was made, it is difficult to assess the actual contribution of the graphene support.

**Figure 3:**
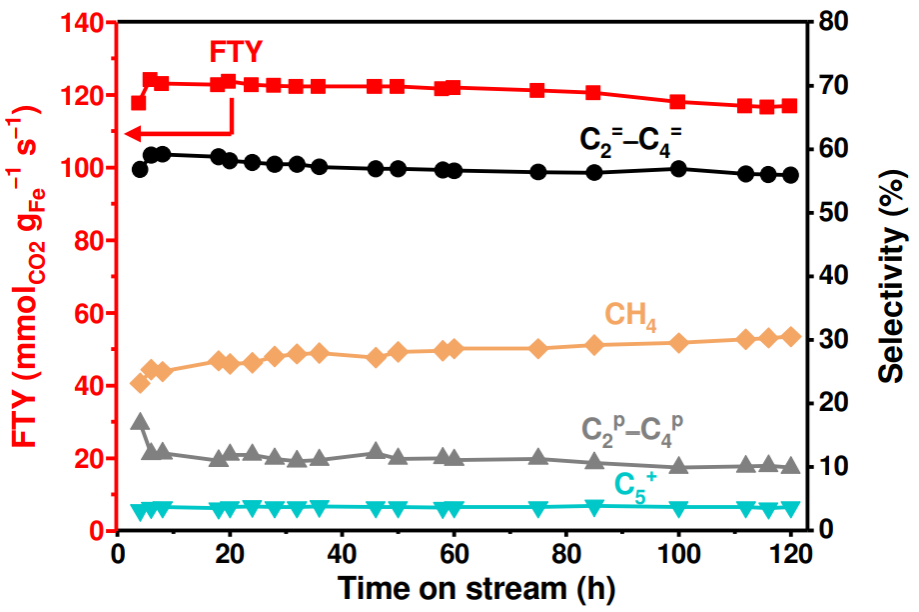
FTY and hydrocarbons selectivity in CO_2_−FTO over the FeK(1.5)/Gr(3D) catalyst (0.15 g catalyst, H_2_/CO_2_ = 3, *T* = 340 °C, *p* = 20 bar, space velocity of 26 L h^−1^ g^−1^) (reprinted with permission from Ref. [45] © 2018, American Chemical Society Inc., Washington, DC, USA).

Iron catalysts promoted with potassium and deposited on 3D graphene (FeK/Gr(3D)) were tested for CO_2_ direct hydrogenation to light olefins (CO_2_-FTO) [45]. The support presents a 3D mesoporous–macroporous structure (22 nm and 50–70 nm, respectively), which provides good premises for the confinement and stabilization of catalytically active iron carbide nanoparticles. The K promotion significantly increases the adsorption and activation of both CO_2_ and H_2_, but also contributes to the carburization of iron and the generation of K-promoted iron carbide active sites for CO_2_-FTO. It was found that for Fe content of 20 wt.%, the optimum K content is 1.5 wt.%. The reaction conditions were selected to match the typical conditions for CO-FTO. The reaction products were a mixture of CO and hydrocarbons: C_2_–C_4_ olefins, C_2_–C_4_ paraffins, CH_4_, and C_5_^+^ (see the hydrocarbons distribution in Figure 3). The CO selectivity is around 40%. The K promotion drastically influences the reaction selectivity: CH_4_ and paraffins selectivity were cut more than half, and the selectivity of olefins increased more than 3 times for FeK(1.5)/Gr(3D), compared to Fe/Gr(3D). The results obtained using FeK(1.5)/Gr(3D) in CO_2_-FTO were among the best obtained using iron catalysts (as reported at the time of their publication). To demonstrate the superiority of the 3D graphene support, two other carbon-supported catalysts with similar Fe and K content were identically prepared using activated carbon (AC), and rGO as supports. The results expressed both in hydrocarbon yield per g of Fe (FTY), but especially in light olefin yield (FTY^=^), were significantly better for FeK(1.5)/Gr(3D) (Table 4). In the case of AC, this lower activity was explained by: (i) the lower dispersion of metal NPs on AC due to the lower dimensions of the pores, combined with the large NPs size, but also by the almost absent oxygenated groups, which act as anchors for NPs; (ii) the presence of meso-macropores in Gr(3D), which facilitates the escape of light olefins from the catalyst, thus avoiding their over-hydrogenation, a route that the micro-mesoporous structure of AC cannot provide. It is interesting that the 2D rGO supported catalysts perform better in reaction than AC supported ones, sustaining the superiority of graphene over other carbon supports. Nevertheless, the performances of FeK(1.5)/rGO were lower than of FeK(1.5)/Gr(3D), which was completely attributed to the confining effect of the 3D support over the 2D one, as demonstrated by the enhanced stability of the NPs size in the reaction observed for FeK(1.5)/Gr(3D) compared to FeK(1.5)/rGO.

The direct transformation of CO_2_ into gasoline range hydrocarbons is another very promising application with high potential for future development. Combining FeK1.5/Gr(3D) with the acidic HZSM-5 zeolite (SiO_2_/Al_2_O_3_ molar ratio = 50) in a dual-layer mode, a catalyst with very interesting olefination-aromatization characteristics was obtained [46]. The working mechanism of this dual catalyst consists of olefination of CO_2_ on FeK1.5/Gr(3D) as described above, followed by aromatization of light olefins over the acidic sites of the zeolite. The catalyst composition (zeolite type, FeK1.5/Gr(3D)/zeolite ratio; SiO_2_/Al_2_O_3_ ratio), and reaction conditions (temperature, pressure, space velocity) were optimized, resulting in a combination with high efficiency and versatility for CO_2_ transformation into aromatics (Table 4). However, the role of graphene support in the aromatization part of the process was not investigated.

## 6. CO_2_ Reduction to CO

CO_2_ transformation into CO and water, in the presence of hydrogen, is known as the Reverse Water Gas Shift Reaction (RWGS), and it is a very useful process in the context of further CO utilization in the Fischer–Tropsch reaction to produce synfuels and chemicals. The advantage is that, in this case, the mature F–T technology can work more environmentally friendly than now, using CO obtained from CO_2_ (negative CO_2_ footprint), and green hydrogen.

N-dopped graphene-supported catalysts having Fe-Co as an active phase were tested in the RWGS reaction [47]. The support was selected due to its previously proved capacity to disperse and stabilize single atom and/or small metal clusters (CLs) of subnanometric dimensions on its surface [48,49]. The declared aim of the study was to investigate the influence of metal particle size on the activity, and especially the selectivity of these catalysts for RWGS to the detriment of methanation, or higher hydrocarbons production. The authors used an original catalyst preparation method developed in their group, which consists of the impregnation of highly porous chitosan aerogel microspheres with metal salts solution, dehydration, and drying under supercritical CO_2_, followed by pyrolysis in Ar at 900 °C. Chitosan is the simultaneous provider of both C and N; an N dopped defective graphene supported catalyst is thus the result of this preparation process. The metal ions are reduced during thermal treatment due to the reductive conditions of the environment. Two series of catalysts were prepared: (i) one with low metal loading (less than 0.2 wt.%), and Fe/Co ratio increasing from 0.44 to 1.26, which contain only very small metal clusters, less than 1 nm in size; and (ii) the second catalysts series with a metal loading of about two orders of magnitude higher than in the first series, and a Fe/Co ratio in the same range (from 0.62 to 1.98). In these catalysts, the metals most likely appear as Fe-Co alloy NPs in the range of 4.8–2.6 nm (the size decreases with decreasing Fe content). Upon testing in RWGS in the 300–500 °C temperature range and 10 bar, the samples having metal CLs were remarkably more active and selective for CO than those having metal NPs (Table 4). For example, at 450 °C approximately 20% higher CO_2_ conversion was obtained for the metal CLs sample compared to the NPs sample with similar Fe-Co composition, given that the first one contains two orders of magnitude less metals. It should be noted that, in all cases, the main component of the reaction products mixture was CO. Except for CO, only CH_4_ and small amounts of propane and butane were detected.

The role of graphene support in this study was to disperse and stabilize the metal clusters. Alongside carbon vacancies and lattice defects, the N doping provides the defective sites for metal clusters to anchor on the support through strong interactions. The preparation method also contributes to the very good dispersion of metal salts on the highly porous chitosan precursor. All these provide a catalyst support that avoids the agglomeration of otherwise unstable metal CLs, with very important effects on the catalytic activity and selectivity.

## 7. Conclusions

Graphene-based materials proved to be suitable supports for obtaining active, selective, and stable catalysts for CO_2_ hydrogenation. If the support is shaped in 3D form, the catalytic performances are improved due to the synergy between the graphene component and the mesoporous structure.

For CO_2_ hydrogenation to methanol, the graphene-containing catalysts are promising to be further developed due: (i) to the very good selectivity results, and (ii) to the relatively lower content of graphene in the oxidic multicomponent material, which increases the economic viability of such a catalyst.

The results obtained in the hydrogenation of CO_2_ to FA are especially promising, being comparable in terms of turn-over number (TON) and turn-over frequency (TOF) to the ones obtained by homogeneous catalysis. In addition, this type of application does not necessarily require industrial size reactors, as the hydrogen storage-release cycle may be needed at different scales, so that graphene catalysts can be an economically viable option.

For CO_2_ to olefins, the results obtained using Gr(3D) supported catalysts are among the best reported, due to the high selectivity of these catalysts for olefins.

Due to its special structure, N-dopped graphene support can anchor and stabilize very small metal nanoclusters (less than 1 nm), resulting in a catalyst with very good properties for RWGS. Such catalysts, containing very small metal clusters, are promising to be further developed for CO_2_ valorization to other products—not only syngas.

Future investigations are needed for the elucidation of the graphene’s support role in CO_2_ hydrogenation. Most of the papers acknowledge graphene’s role in dispersing and stabilizing the metal nanoparticles, but further investigations are needed to propose reaction mechanisms. For CO_2_ hydrogenation to FA instead, many theoretical studies predicted reaction mechanisms for metal clusters or metal single atoms deposited on graphenes, so future experimental studies are needed to confirm these predictions.

## Figures and Tables

**Table 1:** Graphene-based catalysts for CO_2_ hydrogenation to methane.

Catalysts	Preparation Conditions	Properties of the Catalyst	Reaction Conditions	Results	Ref.
**Ni/rGO** 20 wt.% Ni	Ni/rGO by impregnation on rGO + calcination at 400 °C (Ar) rGO by hydrazine reduction of GO	S_t_ = 321 m^2^/g NiNPs size = 7.7 nm	In situ reduction at 400 °C in H_2_ for 4 h; H_2_/CO_2_ = 4:1 *T* = 250–450 °C *p* = atmospheric GHSV = 36,000 mL·h^−1^·g^−1^	At 350 °C CO_2_ conversion = 78.4% CH_4_ selectivity ~100% CH_4_ yield ~79%	[11]
**Ni/CeO_2_-rGO** 20 wt.% Ni 5 wt.% Ce	Ni/CeO_2_-rGO by impregnation on rGO; + calcination at 400 °C (Ar); rGO by hydrazine reduction of GO	S_t_ = 293 m^2^/g NiNPs size = 6.1 nm	In situ reduction at 400 °C in H_2_ for 4 h; H_2_/CO_2_ = 4:1 *T* = 250–450 °C *p* = atmospheric GHSV = 36,000 mL·h^−1^·g^−1^	At 350 °C CO_2_ conversion = 84.5% CH_4_ selectivity ~100% CH_4_ yield~82%	[11]
**Ni/rGO** Ni:10; 15; 20 wt.%	Wet impregnation on commercial rGO; calcination at 400 °C; reduction in (H_2_ + Ar) at 500 °C	For Ni(15%)/rGO S_t_ = 140 m^2^/g NiNPs size = 4.6 nm ~36 graphitic layers	Reaction in liquid phase (dodecane) 1.25% (*w/v*) H_2_/CO_2_ = 4:1 *T* = 240 °C *p* = 10 bar Reaction time: 2 h	For Ni(15%)/rGO CO_2_ conversion = 55.3% CH_4_ selectivity ~100% STY* = 24.9 mg g_cat_^–1^ h^–1^	[12]
**Ni/CNF-FLG** Ni:10; 20; 40 wt.%	Ni/CNF-FLG by impregnation + reduction at 350 °C in H_2_; CNF-FLG by CVD on Ni/FLG; FLG by exfoliation of commercial expended graphite;	For Ni(10%)/CNF-FLG S_t_ = 172 m^2^/g NiNPs size = 5–10 nm ~7–9 graphitic layers	In situ reduction at 350 °C in H_2_ for 1 h; Inductive heating H_2_/CO_2_ = 4:1 *T* = 280 – 420 °C *p* = atmospheric GHSV = 60,000–180,000 h^−1^	At 360 °C CO_2_ conversion = 85% CH_4_ selectivity ~100%	[13]
**Ni(18 wt.%)/GA** GA = graphene aerogel	Solvothermal synthesis from GO, Ni(NO_3_)_2_ and NH_3_; 180 °C; 12 h	S_t_ = 203 m^2^/g NiNPs size = 11 nm	In situ reduction at 450 °C in H_2_ for 2 h H_2_/CO_2_ = 4:1 *T* = 250–450 °C *p* = atmospheric GHSV = 36,000 h^−1^	At 350 °C CO_2_ conversion = 80% CH_4_ selectivity ~95% CH_4_ yield ~76%	[14]
**NGQDs/Al_2_O_3_** (NGQDs—Nitrogen doped graphene quantum dots) NGQDs: 0.8; 1 and 3 wt.%	NGQDs from GO + DMF at 200 °C (in autoclave) for 10 h; catalysts are obtained by impregnation	QDs of 1–2 C layers and 2–3 nm size; N mostly in pyridinic configuration at the exposed edge sites; N content around 6%.	H_2_/CO_2_ = 4:1 *T* = 100–450 °C *p* = 10 bar GHSV = 18,000 mL·h^−1^·g^−1^	very similar results for all three NGQDs content; At 400 °C CO_2_ conversion ~65% CH_4_ selectivity ~55%	[15]

*STY—space time yield (grams methane/catalyst weight/time).

**Table 2:** Graphene-based catalysts for CO_2_ hydrogenation to methanol.

Catalysts	Properties of the Catalyst	Reaction Conditions	Ref.
**CuO-ZnO-Al_2_O_3_-rGO_x_** x: 5; 10 and 15 wt.%	For CuO-ZnO-Al_2_O_3_-rGO(10 wt.%) S_t_ = 147 m^2^/g S_Cu_ = 12.6 m^2^/g_cu_ Cu dispersion = 9.4%	Reduction in H_2_; 280 °C; 6 h H_2_/CO_2_ = 3:1 *T* = 250 °C *p* = 30 bar GHSV = 12,000 mL·g^−1^·h^−1^	[30]
**CuO-ZnO-Al_2_O_3_-N-rGO_x_** x = 7; 10; 13 and 16 wt.%	For CuO-ZnO-Al_2_O_3_-N-rGO(10 wt.%) S_t_ = 102 m^2^/g S_Cu_ = 58.3 m^2^/g Cu dispersion = 8.6%	Reduction in (H_2_ + N_2_); 300 °C; 6 h H_2_/CO_2_ = 3:1 *T* = 200 °C *p* = 30 bar W/F = 10 g·h·mol^−1^	[28]
**CuO-ZnO-ZrO_2_-rGO_x_** x = 0.5; 1; 2.5; 5 and 10 wt.% of GO	For CuO-ZnO-ZrO_2_-GO(1 wt.%) S_t_ = 145 m^2^/g S_Cu_ = 25.3 m^2^/g Cu dispersion = 11.2% actual GO wt.% = 1.4	Reduction in H_2_; 300 °C; 4 h H_2_/CO_2_ = 3:1 *T* = 200–280 °C *p* = 20 bar	[31]
**CuO-ZnO-ZrO_2_-Al_2_O_3_-rGO** mixed oxides: 20 wt.%	S_t_ = 125 m^2^/g S_Cu_ = 1.9 m^2^/g	Reduction in (H_2_ + N_2_); 300 °C; 2 h H_2_/CO_2_ = 3:1 *T* = 200–280 °C *p* = 10–20 bar GHSV = 6075–10,935 h^−1^	[32]
**CuO-ZnO-N-rGO** 10 wt.% CuO-ZnO Cu:Zn = 1:1	S_t_ = 80 m^2^/g Cu oxidation state is close to 0 for the catalyst reduced 90 min in-situ.	Reduction in H_2_; 300 °C; 30–180 min H_2_/CO_2_ = 3:1 *T* = 250 °C *p* = 15 bar GHSV = 2400 h^−1^	[27]
**CuO-ZnO-N-rGO(3D)** 15 wt.% CuO-ZnO Cu:Zn = 1:1 N-r-GO with 3D structure	For N-rGO catalyst reduced with urea S_t_ = 110 m^2^/g oxideNPs size = 7–8 nm	Reduction in H_2_; 300 °C; 1.5 h H_2_/CO_2_ = 3:1 *T* = 250 °C *p* = 15 bar GHSV = 2444 h^−1^	[29]
**CuO-ZnO-N-rGO** 5; 10; 20 and 30 wt.% CuO-ZnO Cu:Zn = 1:1	For the catalyst with 10 wt.% oxides content S_t_ = 83 m^2^/g	Reduction in H_2_; 350 °C; 1.5 h H_2_/CO_2_ = 3:1 *T* = 200–300 °C *p* = 15 bar GHSV = 2400 h^−1^	[26]
**CuO-ZnO-rGO(3D)** 5; 10; 15 and 20 wt.% CuO-ZnO Cu:Zn = 1:1 r-GO with 3D structure	For the catalyst with 15 wt.% CuO-ZnO and rGO reduced at 140 °C S_t_ = 140 m^2^/g oxideNPs size = 7–8 nm oxidation states of Cu and Zn for all catalysts are close to Cu^0^ and Zn^2+^	Reduction in H_2_; 350 °C; 1.5 h H_2_/CO_2_ = 3:1 *T* = 250 °C *p* = 15 bar GHSV = 2444 h^−1^	[24]
**Cu-ZnO-rGO** Cu:Zn = 1:1	Cu crystallite size = 37 nm	H_2_/CO_2_ = 3:1 *T* = 200–240 °C *p* = 30 bar WHSV = 6.6 h^−1^	[25]
**Cu-ZnO-rGO** from HKUST-1 Cu:Zn = 1.2:1	Cu crystallite size = 27 nm CuNP size = 25 nm	H_2_/CO_2_ = 3:1 *T* = 200–240 °C *p* = 30 bar WHSV = 6.6 h^−1^	[25]
**Cu-Ni-rGO** metal-support mass ratio = 1:10 Cu:Ni = 2:1	bimetallic Cu-Ni NPs of 20 nm Cu—mix of Cu^0^, Cu^+^ and Cu^2+^ Ni—only Ni^2+^ the presence of Ni promotes the Cu reduction	Reduction in (H_2_ + N_2_); 270 °C; 2 h H_2_/CO_2_ = 3:1 *T* = 225 °C *p* = 40 bar GHSV = 2500 h^−1^	[33]
**Cu-Ni-N-rGO** metal-support mass ratio = 1:10 Cu:Ni = 2:1	bimetallic Cu-Ni NPs of 50 nm Cu—mix of Cu^0^, Cu^+^ and Cu^2+^ Ni—only Ni^2+^ the presence of Ni inhibits the reduction of Cu	Reduction in (H_2_ + N_2_); 270 °C; 2 h H_2_/CO_2_ = 3:1 *T* = 225 °C *p* = 40 bar GHSV = 2500 h^−1^	[33]

W/F—catalyst weight/flow rate of feed gas; STY—space time yield (grams methanol/catalyst/time).

**Table 3:** Graphene-based catalysts for CO_2_ hydrogenation to formic acid.

Catalysts	Reaction Conditions	Results	Ref.
**Pd_3_Ni_5_/CNT-rGO** PdNi NPs size: 4 nm (impregnation + hydrazine reduction)	H_2_/CO_2_ = 1 *t* = 15 h *T* = 40–70 °C *p* = 10–80 bar	FA yield: 1.35 mmol at 40 °C and 40 bar TON = 5.4 TOF = 1 × 10^−4^ s^−1^	[35]
**Pd_x_Au_(1-x)_/PDA-rGO** PdAu NPs size: 1.6 and 5 nm Metal loading: 5–27.6 wt.% (impregnation + NaBH_4_ reduction)	*t =* 2–24 h *T* = 30 °C, 50 °C, 80 °C *p* = 10–70 bar	For Pd_0.5_Au_0.5_/PDA-rGO Yield: 94% at 50 °C, 50 bar, in 16 h	[36]
**Pd_0.49_Au_0.51_/rGO** PdAu NPs size: 2.7 nm Metal loading: 15.8 wt.% (impregnation + NaBH_4_ reduction)	*t* = 16 h *T* = 50 °C *p* = 50 bar	Yield: 85%	[36]

PDA = phenylenediamine; TON—turnover number; TOF—turnover frequency.

**Table 4:** Graphene-based catalysts for CO_2_ reduction to high hydrocarbons and to CO.

Catalysts	Preparation Conditions	Catalyst’s Properties	Reaction Conditions	Results	Ref.
**Co/rGO(3D)** Co content 20 wt.%	Solvothermal reduction of Co(acac)_2_ + GO in DMF at 180, 200, and 220 °C for 12 h	Wire-like hexagonal phase CoNPs and 3D rGO obtained only for sample reduced at 220 °C S_t_ = 235 m^2^/g CoNPs size = 29 nm	In situ reduction at 400 °C in H_2_ for 0.5 h H_2_/CO_2_ = 4:1 *T* = 200–300 °C *p* = 1 bar	Reaction products: C_1_–C_5_ paraffins and olefins CO_2_ conversion ~ 12% (for catalyst prepared at 220 °C)	[44]
**FeK/Gr(3D)** Fe content 18 wt.% K content 1.5 wt.%	FeK/Gr(3D) by sequential impregnation of Fe and K Gr(3D) by CVD from CO on Li_2_O	S_t_ = 129 m^2^/g Fe_3_O_4_NPs size = 10 nm	H_2_/CO_2_ = 3 *t* = 2 h *T* = 340 °C *p* = 20 bar GHSV = 26 lg^−1^ h^−1^	CO_2_ conversion 46% FTY 123 μmol_CO2_ g_Fe_^–1^s^–1^ FTY^=^ 73 μmol_CO2_ g_Fe_^–1^s^–1^ olefin selectivity: 59%	[45]
**FeK/rGO** Fe content 20 wt.% K content 1.5 wt.%	FeK/rGO by sequential impregnation of Fe and K rGO by hydrazine reduction of GO	Fe_3_O_4_ NPs size = 13 nm	H_2_/CO_2_ = 3 *T* = 340 °C *p* = 20 bar GHSV = 26 lg^−1^ h^−1^	CO_2_ conversion 37% FTY^=^ 76 μmol_CO2_ g_Fe_^–1^s^–1^ FTY^=^ 43 μmol_CO2_ g_Fe_^–1^s^–1^ olefin selectivity: 56%	[45]
**FeK/Gr(3D)/HZSM-5** Fe content 18 wt.% K content 1.5 wt.%	Pressing the two layers of FeK/Gr(3D) and HZSM-5, followed by crushing and sieving	FeK/Gr(3D): HZSM-5 = 1:1	H_2_/CO_2_ = 3 *T* = 340 °C *p* = 20 bar GHSV = 26 lg^−1^ h^−1^	CO_2_ conversion 35% STY_aro_ 11.8 μmol_CO2_ g_cat_^–1^ s^–1^ aromatic selectivity: 68%	[46]
**Fe_x_-Co_y_/(N)Gr ** low metal content (<0.2 wt.%) N content 5 wt.%	Metal impregnation on chitosan aerogel + pyrolysis (concomitant metal reduction)	Fe/Co ratio: 1.26–0.44 MeCLs size < 1 nm	H_2_/CO_2_ = 7 *T =* 300–500 °C *p =* 10 bar GHSV = 600 h^−1^	Best results for Fe/Co = 1.26; (Fe + Co) = 0.095 wt.% CO_2_ conversion = 56% (500 °C) CO selectivity = 98%	[47]
**Fe_x_-Co_y_/(N)Gr ** high metal content (6–16 wt.%) N content 5 wt.%	Metal impregnation on chitosan aerogel + pyrolysis (concomitant metal reduction)	Fe/Co ratio: 1.98–0.62 MeNPs size: 2.6–4.8 nm	H_2_/CO_2_ = 7 *T* = 300–500 °C *p* = 10 bar GHSV = 600 h^−1^	Best result for Fe/Co = 0.62; (Fe + Co) = 6.6 wt.% CO_2_ conversion = 40% (500 °C) CO selectivity ~ 85%	[47]

FTY—iron time yield of hydrocarbons; FTY^=^—iron time yield of light olefins; STY_aro_—productivity of aromatics.

## Abbreviations

AC	activated carbon
CLs	clusters
CNF	carbon nanofibers
CNT	carbon nanotubes
CO_2_-FTO	CO_2_ direct hydrogenation to light olefins
CVD	chemical vapor deposition
FLG	few layers graphene
FTY	iron time yield of hydrocarbons
FTY^=^	iron time yield of light olefins
GHSV	gas hourly space velocity
GO	graphene oxide
Gr	graphene
NPs	nanoparticles
N-rGO	N-dopped reduced graphene oxide
rGO	reduced graphene oxide
RWGS	reverse water gas shift
STY	space time yield
STY_aro_	productivity of aromatics
TOF	turnover frequency
TON	turnover number
TOS	time on stream
WHSV	weight hourly space velocity
